# Estimation of knee joint movement using single-channel sEMG signals with a feature-guided convolutional neural network

**DOI:** 10.3389/fnbot.2022.978014

**Published:** 2022-10-25

**Authors:** Song Zhang, Jiewei Lu, Weiguang Huo, Ningbo Yu, Jianda Han

**Affiliations:** ^1^College of Artificial Intelligence, Nankai University, Tianjin, China; ^2^Tianjin Key Laboratory of Intelligent Robotics, Nankai University, Tianjin, China; ^3^Institute of Intelligence Technology and Robotic Systems, Shenzhen Research Institute of Nankai University, Shenzhen, China

**Keywords:** single-channel sEMG signals, human-robot interaction, joint movement estimation, level walking, feature-guided convolutional neural network (FG-CNN)

## Abstract

Estimating human motion intention, such as intent joint torque and movement, plays a crucial role in assistive robotics for ensuring efficient and safe human-robot interaction. For coupled human-robot systems, surface electromyography (sEMG) signal has been proven as an effective means for estimating human's intended movements. Usually, joint movement estimation uses sEMG signals measured from multiple muscles and needs many sEMG sensors placed on the human body, which may cause discomfort or result in mechanical/signal interference from wearable robots/environment during long-term routine use. Although the muscle synergy principle implies that it is possible to estimate human motion using sEMG signals from even one signal muscle, few studies investigated the feasibility of continuous motion estimation based on single-channel sEMG. In this study, a feature-guided convolutional neural network (FG-CNN) has been proposed to estimate human knee joint movement using single-channel sEMG. In the proposed FG-CNN, several handcrafted features have been fused into a CNN model to guide CNN feature extraction, and both handcrafted and CNN-extracted features were applied to a regression model, i.e., random forest regression, to estimate knee joint movements. Experiments with 8 healthy subjects were carried out, and sEMG signals measured from 6 muscles, i.e., vastus lateralis, vastus medialis, biceps femoris, semitendinosus, lateral or medial gastrocnemius (LG or MG), were separately evaluated for knee joint estimation using the proposed method. The experimental results demonstrated that the proposed FG-CNN method with single-channel sEMG signals from LG or MG can effectively estimate human knee joint movements. The average correlation coefficient between the measured and the estimated knee joint movements is 0.858 ± 0.085 for LG and 0.856 ± 0.057 for MG. Meanwhile, comparative studies showed that the combined handcrafted-CNN features outperform either the handcrafted features or the CNN features; the performance of the proposed signal-channel sEMG-based FG-CNN method is comparable to those of the traditional multi-channel sEMG-based methods. The outcomes of this study enable the possibility of developing a single-channel sEMG-based human-robot interface for knee joint movement estimation, which can facilitate the routine use of assistive robots.

## 1. Introduction

Surface electromyography (sEMG) has been extensively used to ensure accurate and safe human-robot interaction (HRI) in robotic devices for rehabilitation or performance enhancement (Nam et al., 2014; Spanias et al., 2016; Caulcrick et al., 2021). Regarding the sEMG-based HRI, one crucial issue is to estimate human motion intention (e.g., intended joint movements) from the sEMG signals (Ding et al., 2017; Bi et al., 2019; Lu et al., 2019). Due to the sEMG signal with the characteristics of preceding the corresponding motion by 20–100 ms and containing neuromuscular control information, the sEMG-based motion estimation benefits in achieving a more natural and fluent HRI and can differentiate how much of the motion is caused by muscles: a unique advantage compared with the inertial measurement unit (IMU)/optical-based method (Xiong et al., 2021). Recently, many approaches have been proposed to estimate the human joint movements based on multi-channel sEMG signals, such as adaptive hybrid classifier for hand gesture recognition (Ding et al., 2019) and Hill-based method or deep learning method for joint movement prediction (Fleischer and Hommel, 2008; Wang et al., 2021; Zhong et al., 2022).

Although multi-channel sEMG signals can provide rich information and contribute to estimate the corresponding joint movement accurately, using multi-channel sEMG has some practical limitations: first, collecting multi-channel sEMG subjects is subject to some limitations, such as weakness or spasticity of one or more specific muscles and mechanical/signal interference between sEMG sensors and wearable robots/environment (e.g., sitting on a chair); second, increasing the number of physical channels would increase the system complexity, making it difficult to deploy, as well as increase the power consumption (He et al., 2019). The above drawbacks limit the routine use of sEMG-based assistive robots. Therefore, it is important to investigate the estimation of human motion using sEMG signals from fewer muscles or even a single muscle.

Recently, some related studies on hand gesture identification (Kumar et al., 2013), upper limb movement recognition (Shao et al., 2020), terrain identification (Gupta and Agarwal, 2019), and lower limb movement recognition (Wei et al., 2022) used single-channel sEMG signals. However, the existing studies mainly focused on recognizing discrete motion modes rather than estimating continuous joint movements. Compared with discrete modes, continuous joint movements can enable simultaneous and proportional control (Bao et al., 2021), realizing more effective and safer HRI for rehabilitation and assistive robots and orthoses. To the best of our knowledge, few studies demonstrated an accurate joint movement estimation method based on single-channel sEMG. According to the muscle synergy principle, which is widely accepted as a constitutional function unit of the central neural systems that control muscles in groups (d'Avella et al., 2003; Jiang et al., 2014; Dwivedi et al., 2020; Kubota et al., 2021), a group of related muscles' activities have certain common components or patterns, which enables the possibility of estimating joint movements using sEMG signals from one or fewer muscles. Therefore, developing a single-channel sEMG-based continuous joint movement estimation method has great potential for facilitating the routine use of assistive robots.

Compared to recognizing motion modes, it is more challenging to accurately estimate continuous joint movements using single-channel sEMG signals due to the limited muscular information. To guarantee an accurate and robust estimation of human joint movement, it is crucial to extract muscular information from single-channel sEMG signals adequately. There are two main ways of extracting muscular information: One is directly computing handcrafted features using mathematical equations (Phinyomark et al., 2012; Thongpanja et al., 2016) and another one is to extract learning features by deep learning, e.g., convolutional neural network (CNN). The learning features may complement the handcrafted features (Atzori et al., 2016; Phinyomark and Scheme, 2018; Côté-Allard et al., 2020). Therefore, it is possible to obtain relatively adequate muscular information from single-channel sEMG by fusing the handcrafted and learning features.

In this study, a new feature extraction method, namely feature-guide convolutional neural network (FG-CNN), was proposed to estimate knee joint movements using single-channel sEMG signals. In the proposed FG-CNN, 14 handcrafted features (Wei et al., 2019) were first fed into a fusion layer to guide a traditional CNN in extracting 14 implicit features (i.e., CNN extracted features). The 28 FG-CNN features containing 14 handcrafted features and 14 CNN features were applied to a regression model, e.g., random forest regression, to estimate continuous knee joint movements. To verify the effectiveness of the FG-CNN, the proposed method was respectively evaluated on six kinds of single-channel sEMG signals measured from vastus lateralis (VL), vastus medialis (VM), biceps femoris (BF), semitendinosus (ST), lateral and medial gastrocnemius (LG and MG) for estimating the movements. Meanwhile, the 28 FG-CNN features were respectively compared to 14 handcrafted features and 28 CNN features (extracted by the traditional CNN) on the same regression model. The experimental results show that the proposed FG-CNN method with single-channel sEMG signals from LG or MG can effectively estimate the movement. The FG-CNN features outperform the handcrafted features and CNN features on single-channel sEMG-based movement estimation, suggesting that the FG-CNN features contain more muscular information.

The main contributions of this study are as follows:

1. This is the first study, to our knowledge, to investigate the feasibility of using single-channel sEMG signals to estimate the human knee angles.
2. A new feature extraction algorithm has been developed to extract muscular information from single-channel sEMG adequately and a new scheme is proposed to estimate knee joint angles based on single-channel sEMG signals using FG-CNN and regression models.
3. The effectiveness of the proposed method has been evaluated *via* experiments with eight subjects during walking. sEMG signals from a single muscle, LG or MG, can be used to improve the estimation performance with the proposed method.

## 2. Methods

### 2.1. Feature-guided convolutional neural network

**Figure 1:**
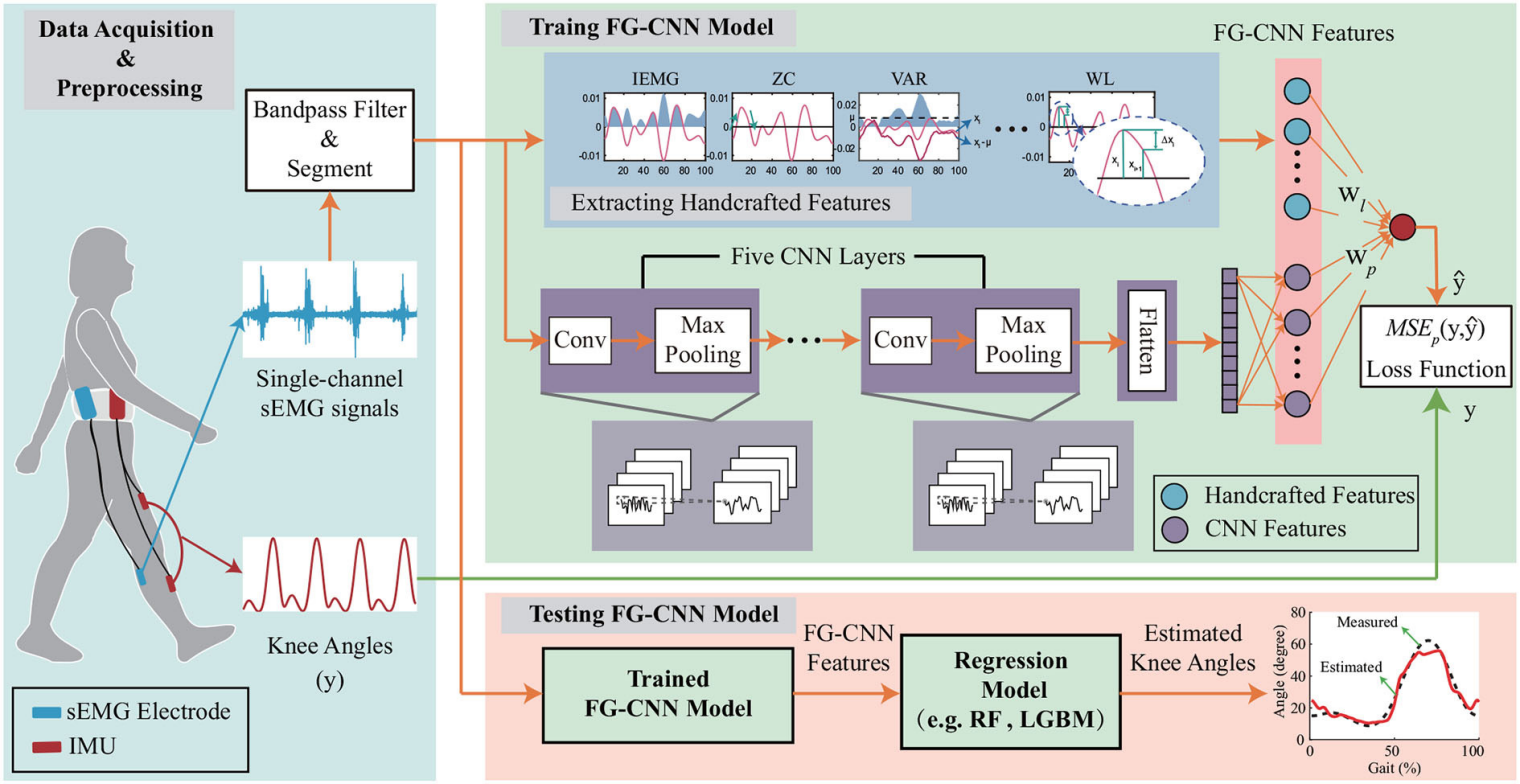
The overall framework of the FG-CNN-based motion estimation. The FG-CNN consists of the operation of extracting handcrafted features, five CNN layers, one flatten layer, and one fusion layer for fusing CNN features and handcrafted features. IMU represents the inertial measurement unit; Conv denotes the convolutional layers; IEMG, VAR, ZC, and WL represent the handcrafted features. The symbols w_l_ and w_p_, respectively, represent the weights of the handcrafted and CNN features in the fusion layer. RF and LGBM represent the random forest model and light gradient boosting machine, respectively.

The proposed FG-CNN was depicted in Figure 1. In the FG-CCN, 14 typical sEMG features, i.e., the handcrafted features, were extracted from the raw sEMG data and fed into a fusion layer of a CNN to guide the CNN in extracting implicit muscular information. Furthermore, both the handcrafted features and the CNN extracted features were connected and used to estimate knee joint movements using a regression model, e.g., random forest regression (RF) and light gradient boosting machine (LGBM).

#### 2.1.1. Handcrafted feature extraction

A method called overlapping analysis windows with a window length of 50 ms and an increment of 20 ms was used to segment the sEMG signals. The vector **x** = {*x*_1_, *x*_2_, …, *x*_*n*_} represents sEMG signal in a window, where *n* is the length of **x**. 14 handcrafted features, including integrated EMG (IEMG), mean absolute value (MAV), mean, root mean square (RMS), variance (VAR), Kurtosis, skewness, zero crossing (ZC), slop sign change (SSC), waveform length (WL), and four auto-regressive (AR) model coefficients are calculated using the overlapping analysis windows (Wei et al., 2019). The above 14 handcrafted features are concatenated as a vector (**p** = {IEMG, MAV, …, AR_4_}) fused into a CNN to extract CNN features.

#### 2.1.2. FG-CNN feature extraction

The CNN feature extraction (see Figure 1) is used to extract implicit muscular information from the input sEMG signal vector **x**. In the CNN structure, five convolution layers had 2, 4, 8, 16, and 32 filters, respectively, where the filters were 5 × 1, 4 × 1, 3 × 1, 2 × 1, and 1 × 1. Max pooling was conducted on 2 × 1 area with a stride of 1. The two fully-connected layers contain 192 and 14 neurons. For each convolutional layer, **x** ∈ ℝ^*L*′ × *D*′^ is defined as the input, where the *L*′ and *D*′ denote the length and the number of channels. Assuming the *D* convolutional kernels **k**, the output of the convolutional layer **y** ∈ ℝ^*L*×*D*^ is described as:


(1) $$\begin{array}{l} y=f(k*x+w), \end{array}$$

where *f* represents an activation function, **w** is a bias parameter vector, and * denotes convolution.

The LeakyReLU nonlinearity (Maas et al., 2013) is applied as the activation function of convolutional layers mentioned in (1), which is defined as:


(2) $$\begin{array}{l} f(x)=\{\begin{array}{ll} x, & \text{if }x>\text{0}\\ ax, & \text{otherwise,} \end{array} \end{array}$$

where *a* is a learnable parameter.

To avoid overfitting the model, batch normalization (BN) (Ioffe and Szegedy, 2015) is applied after each convolutional layer. The convolutional layer is followed by a max-pooling layer with a length of 2, which transforms the outputs of multiple neurons in one layer into a single neuron in the next layer. The input of the pooling layer is the output of the convolutional layer before it, i.e., **y** ∈ ℝ^*L*×*D*^. The output of each pooling layer ${\text{y}}^{\prime }\in {\mathbb{R}}^{\frac{L}{2}\times D}$ is described as:


(3) $$\begin{array}{l} y^{\prime }(i,j)=\max(y(2i-1,j),y(2i,j)), \end{array}$$

where $i=1,2,\ldots \frac{L}{2}$, *j* = 1, 2, …*D*.

The output of the last pooling layer is then flattened into a vector, **l** ∈ ℝ^14 × 1^, which is called CNN features.

A fusion layer is introduced to combine the extracted handcrafted features (**p**) and the CNN extracted features (**l**). In the fusion scheme, both the handcrafted and CNN extracted features are fed into the last fully connected layer to estimate the joint motion ($\hat{\text{y}}$) (see Figure 1). Instead of using the LeakyReLU, the tanh function, which normalizes fused features to [-1,1], is chosen to avoid the blow-up phenomenon. The fusion scheme is defined as follows:


(4) $$\begin{array}{l} \hat{y}=\tanh(w_{p}p+w_{l}l), \end{array}$$

where **w**_**p**_ and **w**_**l**_ refer to the connection weights.

The built FG-CNN model is trained using a mean squared error function (*MSE*_*p*_) as follows:


(5) $$\begin{array}{l} MSE_{p}=\frac{1}{N}\sum_{i=1}^{N}(y(i)-\hat{y}(i){)}^{2}\\ \;=\frac{1}{N}\sum_{i=1}^{N}(y(i)-\tanh(w_{p}p(i)+w_{l}l(i)){)}^{2}, \end{array}$$

where **y** and $\hat{\text{y}}$ denote the measured and estimated knee joint movements, respectively. *N* is the total number of samples.

During the training, Adam algorithm (Diederik and Jimmy, 2015) is utilized to update the weights. Unlike the traditional CNN, the proposed FG-CNN includes the handcrafted features in the weight updating process to guide the CNN to extract features. Correspondingly, the weights of the fusion layer of the FG-CNN are updated in the following steps.

Step 1: Compute the gradients:


(6) $$\begin{array}{l} \{\begin{array}{l} \frac{\partial MSE_{p}}{\partial w_{p}}=\frac{\partial MSE_{p}}{\partial \hat{y}(i)}\frac{\partial \hat{y}(i)}{\partial w_{p}}=-\frac{2p}{N}\sum_{i=1}^{N}(y(i)-\hat{y}(i))(1-\hat{y}{\left(i\right)}^{2})\\ \frac{\partial MSE_{p}}{\partial w_{l}}=\frac{\partial MSE_{p}}{\partial \hat{y}(i)}\frac{\partial \hat{y}(i)}{\partial w_{l}}=-\frac{2l}{N}\sum_{i=1}^{N}(y(i)-\hat{y}(i))(1-\hat{y}{\left(i\right)}^{2}) \end{array} \end{array}$$

Step 2: Update the weights:


(7) $$\begin{array}{l} \left\{ \begin{array}{l} w_{p}\leftarrow w_{p}-\frac{a_{p}+b\frac{\partial MSE_{p}}{\partial w_{p}}}{\sqrt{c_{p}-d{\left(\frac{\partial MSE_{p}}{\partial w_{p}}\right)}^{2}}+\epsilon }\\ w_{l}\leftarrow w_{l}-\frac{a_{l}+b\frac{\partial MSE_{p}}{\partial w_{l}}}{\sqrt{c_{l}-d{\left(\frac{\partial MSE_{p}}{\partial w_{l}}\right)}^{2}}+\epsilon }, \end{array}\right. \end{array}$$

where ϵ denotes the constant, ϵ = 10^−8^. **a**_**p**_, **a**_**l**_, **b**, **c**_**p**_, **c**_**l**_, and **d** are given as follows:


(8) $$\begin{array}{l} \;a_{p}=\frac{\alpha \beta_{1}s_{p}}{1-\beta_{1}^{t}}\;a_{l}=\frac{\alpha \beta_{1}s_{l}}{1-\beta_{1}^{t}}\;c_{p}=\frac{\alpha \beta_{2}r_{p}}{1-\beta_{2}^{t}}\\ c_{l}=\frac{\alpha \beta_{2}r_{l}}{1-\beta_{2}^{t}}\;b=\frac{\alpha (1-\beta_{1})}{\left(1-\beta_{1}^{t}\right)}\;d=\frac{\alpha (1-\beta_{2})}{\left(1-\beta_{2}^{t}\right)}, \end{array}$$

where **s_p_** and **s_l_** denote the first and second order moment vector of handcrafted features, **r_p_** and **r_l_** denote the first and second order moment vector of FG-CNN features, and β_1_, $\beta_{1}^{t}$, β_2_, $\beta_{2}^{t}$ are the exponential decay rates for the moment estimations. α denotes the step-size.

As seen in Equation (7), the updated weights of the fusion layer are updated using both handcrafted and CNN extracted features. With a learning rate of 0.001, the FG-CNN was trained on NVIDIA Quadro P5000 GPU by using the Adam algorithm for 50 epochs in our experiments.

### 2.2. Experimental protocol and data acquisition

Eight healthy subjects (six men and two women, aged 25.13 ± 3.27 years old) participated in the experiments. All experiments were conducted in accordance with the ethical standards encoded in the latest Declaration of Helsinki. Before the experiments, each participant was fully informed of the experimental purpose and procedures and provided their written consent to participate in this study. The experiments were proved by the local ethics committee of Nankai University.

**Figure 2:**
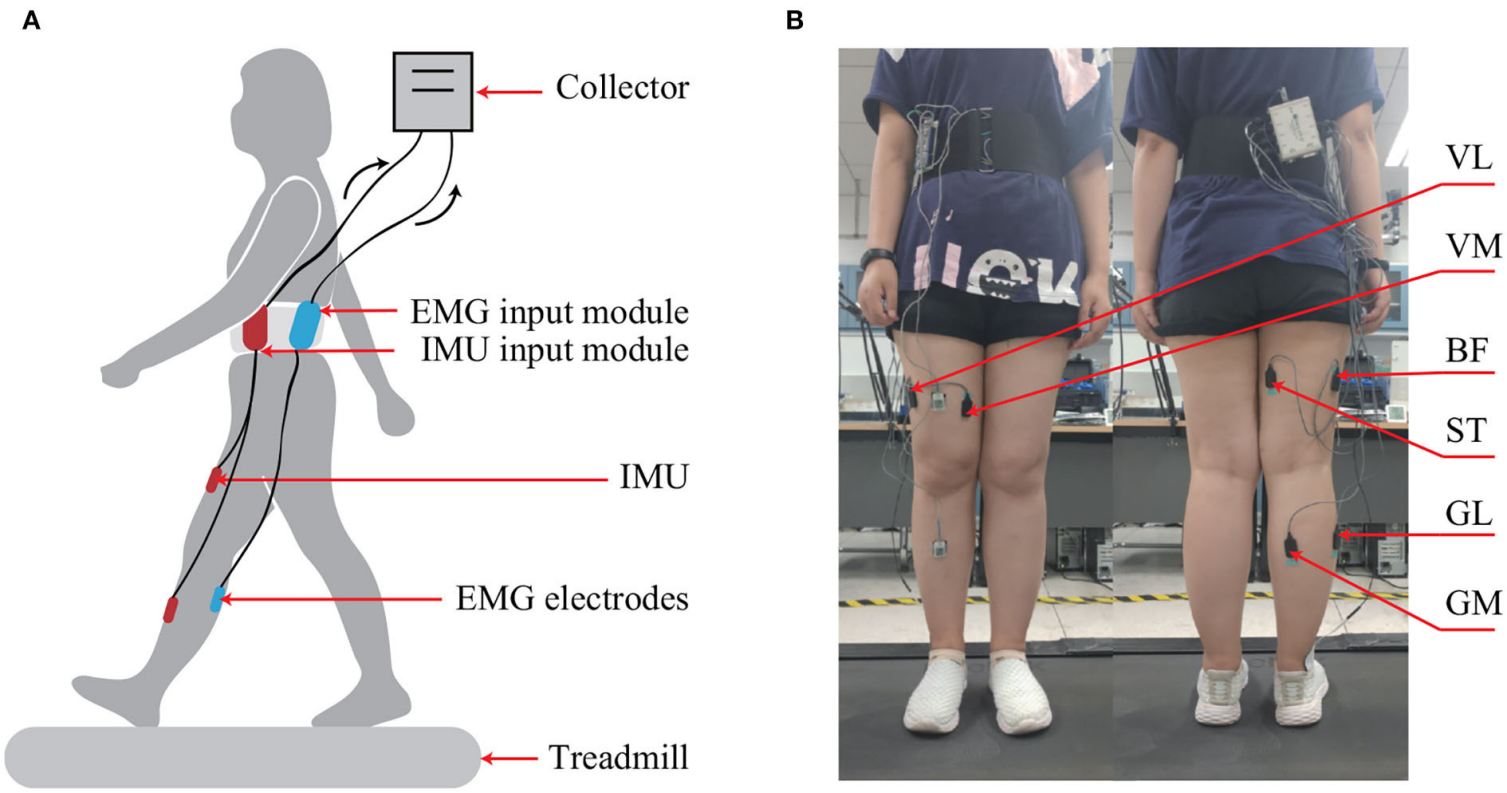
The experimental setup. **(A)** Schematic diagram of the experimental setup. **(B)** The locations of sEMG electrodes.

The experiment scheme is shown in Figure 2. Six channels of sEMG electrodes were respectively placed on six muscles, namely, vastus lateralis (VL), vastus medialis (VM), biceps femoris (BF), semitendinosus (ST), lateral gastrocnemius (LG), and medial gastrocnemius (MG) (Lu et al., 2021), which are relative to the knee joint motion. The data of sEMG were obtained by an acquisition system (Bagnoli, Delsys, MA, USA) under the sampling rate of 5 kHz. At the same time, the data of knee joint angles were also measured using two IMUs with the sampling rate of 100 Hz. Each subject was asked to walk for 1 min with a velocity of 1.25 m/s on the treadmill per trial and perform 11 trials in total with a 3-min rest in between to avoid muscle fatigue. The raw sEMG signals were pre-processed using a Butterworth bandpass filter with cutoff frequencies of 10 Hz and 500 Hz. The sEMG signals measured from the six muscles were tested separately to estimate the knee joint movements. Five cross-validations were used to split the dataset into training data and testing data that are independent of each other. For each time, the proposed FG-CNN model was trained on 80% of data and evaluated on 20% of data.

### 2.3. Evaluation indicators

The estimation performance of the trained FG-CNN was evaluated by using two indicators: normal root-mean-squared error (NRMSE) and correlation coefficients (CC), respectively (Kwon and Kim, 2011; Qing et al., 2022). The NRMSE is used to reflect the deviation between the measured and estimated knee joint angles, in percentage (%) (Zhu et al., 2022). The CC value can reflect the strength of the correlation between the measured and estimated knee joint angles, which is close to 1, which indicates meaning a good match between the measurement and the estimation.

The NRMSE is defined as


(9) $$\begin{array}{l} \text{NRMSE}=\frac{\sqrt{\frac{1}{n}\;\cdot \sum_{i=1}^{n}{\left(y_{i}-{\hat{y}}_{i}\right)}^{2}}}{y_{\max}-y_{\min}}, \end{array}$$

where *y*_*i*_ and ŷ_*i*_ are the measured and estimated knee joint angles, respectively, *n* denotes the total number of samples, and *y*_max_ and *y*_min_ are the maximum and minimum values of the measured angles, respectively.

CC is defined as


(10) $$\begin{array}{l} \text{CC}=\frac{C_{y\hat{y}}}{\sigma_{y}\cdot \sigma_{\hat{y}}}, \end{array}$$

where *C*_*yŷ*_ denotes the covariance between the measured and estimated angles and σ_*y*_ and σ_ŷ_ represent the standard deviation of measured and estimated angles, respectively.

### 2.4. Statistical analysis

Statistical analyses were performed to compare the estimation performances between the proposed FG-CNN and the other compared methods. As the evaluation indicators were not normally distributed, the Kruskal-Wallis test was conducted to compare the different estimation methods with the FG-CNN to identify differences in NRMSE and CC. For all tests, the significance level was set at a *p* < 0.05. Statistical analyses were conducted with MATLAB (MathWorks, Natick, MA, USA).

## 3. Experimental results

### 3.1. Performance of knee joint estimation

To verify the effectiveness of the proposed method in extracting implicit features from single-channel sEMG signals, a comparison study was carried out, in which the handcrafted features, the CNN features, and the FG-CNN features were separately used to estimate the knee joint movements *via* a random forest (RF) regression model (see Figure 1).

The details are given as follows:

1. HF-RF: Fourteen handcrafted features were fed into the RF regression.
2. CNN-RF: Twenty-eight CNN features were used as the inputs of the RF regression.
3. FG-CNN-RF: Twenty-eight FG-CNN features were fed into the RF regression.

**Figure 3:**
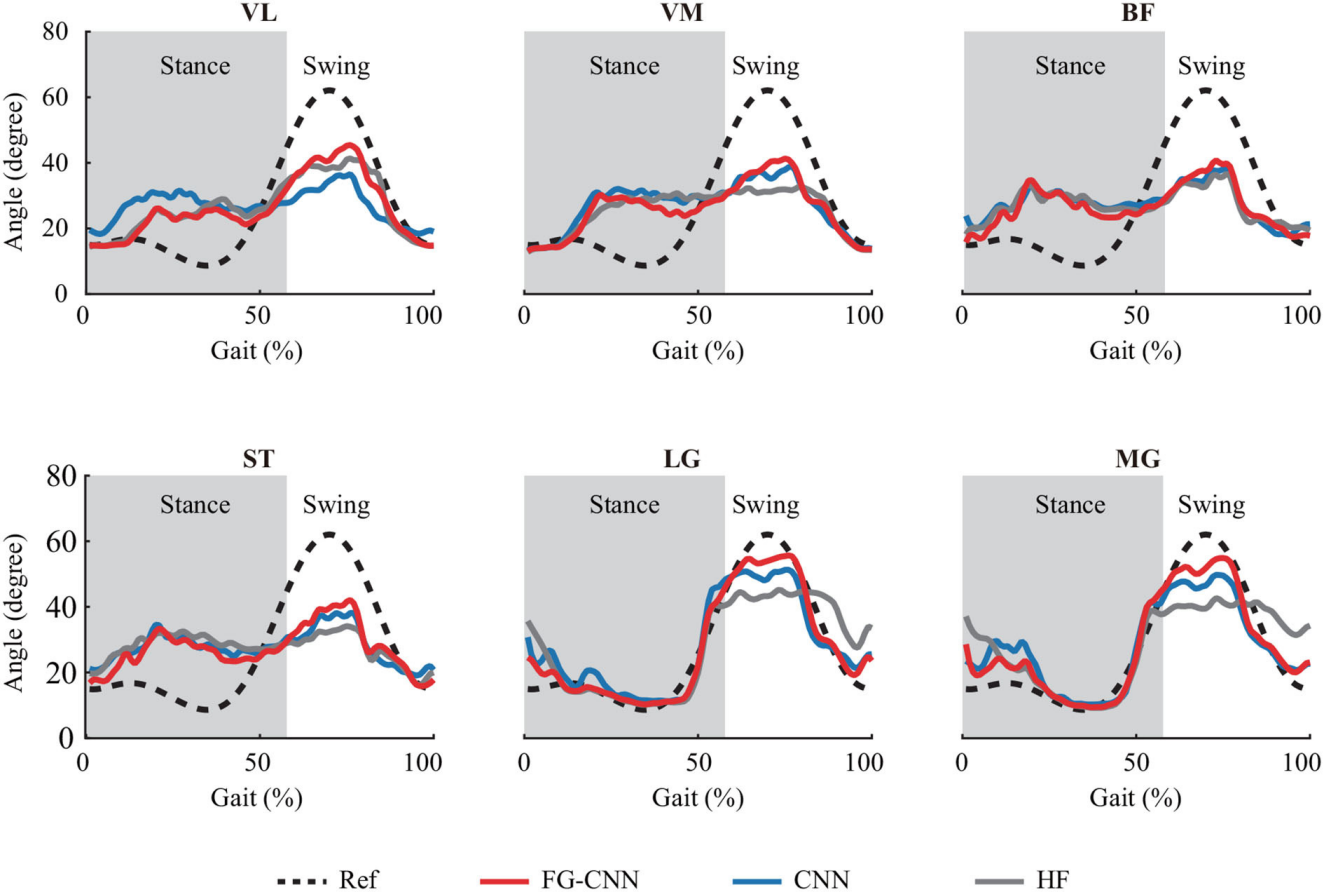
Knee joint angles profiles averaged across 57 strides with subject 2. Dashed and solid lines are reference angles measured by IMUs and estimated angles by FG-CNN-RF, CNN-RF, and HF-RF, using single-channel sEMG signals. The gray shaded area indicates the stance phase.

Figure 3 shows the knee joint angles measured using the IMUs (i.e., the reference) and the ones estimated using the HF-RF, CNN-RF, and FG-CNN-RF. For all approaches, the estimated angles from FG-CNN-RF are closer to the reference than those from HF-RF and CNN-RF. For all muscles, it can also be seen that the estimated knee joint angles using single-channel sEMG signals measured from LG or MG are more accurate than those estimated using sEMG signals from VL, VM, BF, or ST.

**Figure 4:**
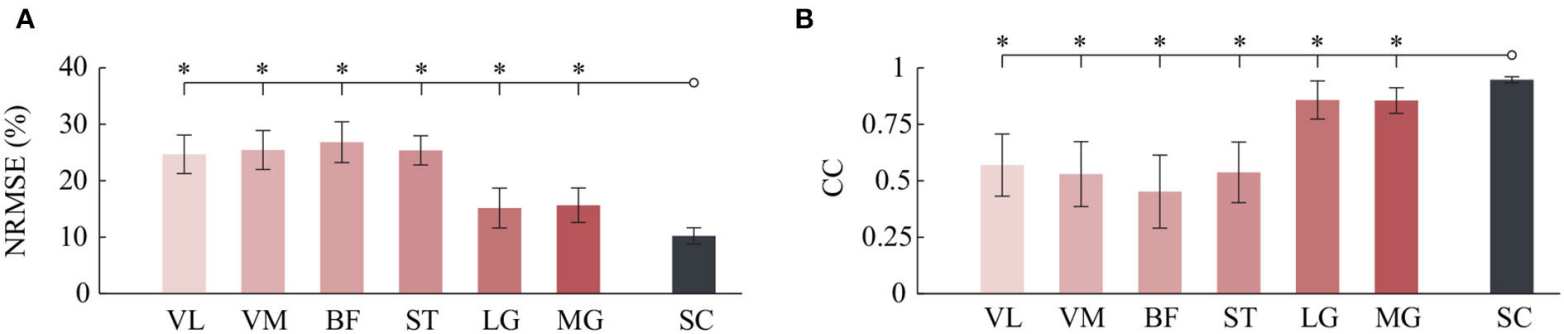
Comparison of angle estimation accuracy between six kinds of single-channel sEMG and six-channel sEMG (SC). **(A)** Comparison of NRMSE. **(B)** Comparison of CC. Bars are means, error bars are standard error of the mean (SEM), and asterisks denote statistically significant differences with respect to the six-channel sEMG (*P* < 0.05).

Meanwhile, to quantitatively evaluate the estimation results, the indicators of NRMSE and CC were used. The NRMSE values using the LG and MG from the FG-CNN-RF were, respectively, 15.2±3.5% (LG) and 15.7±3.1% (MG), and the CC values were, respectively, 0.858 ± 0.085 (LG) and 0.856 ± 0.057 (MG). Although the estimation performance obtained from single-channel sEMG signals (LG or MG) is slightly lower than that from six-channel signals (NRMSE: 10.2±1.4%; CC: 0.948±0.013, shown in Figure 4), the estimation accuracy using the proposed single-channel based method is comparable to the that using six-channel sEMG. The results can be explained by the muscle synergy analysis, in which the muscles are controlled in groups to generate a desired joint movement, and all relevant muscles share some common components.

**Figure 5:**
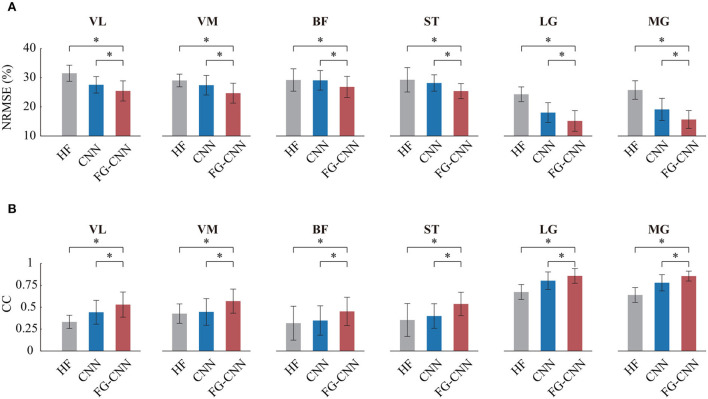
Comparison of angle estimation accuracy by HF, CNN, and FG-CNN. **(A)** Comparison of NRMSE. **(B)** Comparison of CC. Bars are means, error bars are standard error of the mean (SEM), and asterisks denote statistical significance (*P* < 0.05).

For each muscle, the mean NRMSE values of the FG-CNN-RF were lowest and decreased by 15% (VL), 19.2% (VM), 8.1% (BF), 13.3% (ST), 37.6% (LG), and 39.4% (MG) compared to those of the HF-RF and by 11.3% (VL), 9% (VM), 8.6% (BF), 12.1% (ST), 18.8% (LG), and 19.2% (MG) compared to those of the CNN-RF (shown in Figure 5A). The average CC values of FG-CNN-RF were highest, which were increased by 33.4% (VL), 59.3% (VM), 42.4% (BF), 51.7% (ST), 27.3% (LG), and 33.8% (MG) compared to those of the HF-RF, and increased by 32.2% (VL), 24.8% (VM), 34.1% (BF), 42.8% (ST), 7.9% (LG), and 9.8% (MG) compared to those of the CNN-RF (see Figure 5B).

The Kruskal-Wallis test was conducted to identify differences in NRMSE and CC between the case using FG-CNN features and the cases using the other two kinds of features (handcrafted features and CNN features). Both the NRMSE and CC values of FG-CNN had statistically significant differences with respect to handcrafted features and CNN features (see Figure 5). The above results implies that the FG-CNN features contain more muscular information than the handcrafted features or CNN features for ensuring a more accurate estimation of knee joint movement.

### 3.2. Comparison of various regression models

**Figure 6:**
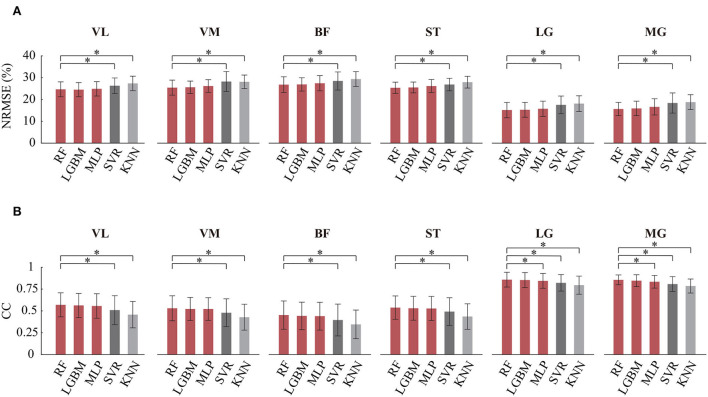
Comparison of angle estimation accuracy on different regression models. RF, random forest regression; LGBM, gradient boosting machine; MLP, multilayer perceptron (MLP); SVR, support vector regression; KNN, k-nearest neighbors regression. **(A)** Comparison of NRMSE. **(B)** Comparison of CC. Bars are means, error bars are standard error of the mean (SEM), and asterisks denote statistically significant differences with respect to the RF (*P* < 0.05).

To further test the effectiveness of the FG-CNN features, five difference regression models were used, including RF regression, light gradient boosting machine (LightGBM), multilayer perceptron (MLP), support vector regression (SVR), and k-nearest neighbors (KNN) regression. The inputs of the five models were FG-CNN features. Figure 6 shows the NRMSE and CC values for knee joint movement estimation using the five regression models. The regression models of RF, LightGBM, and MLP have similar estimation performance with lower NRMSE and greater CC values than the other two models.

The Kruskal-Wallis test was also used to determine if there were differences between the RF and the other four regression models. Table 1 showed the statistical analysis results on CC and NRMSE. For each single-channel sEMG, the NRMSE values of RF significantly decreased compared to these of SVR and KNN. Meanwhile, the CC values of RF significantly increased compared to those of SVR and KNN. The CC values of MLP from LG and MG were different compared to that from the RF (LG: *P* = 0.015; MG: *P* = 0.0015), while no significant differences were found between RF and the other two models (LightGBM and MLP) on NRMSE and CC.

**Table 1:** Statistical analysis results between RF and the other regression models (including LGBM, MLP, SVR, and KNN) on NRMSE and CC.

**Muscle**	**NRMSE**				**CC**			
	**LGBM**	**MLP**	**SVR**	**KNN**	**LGBM**	**MLP**	**SVR**	**KNN**
VL	0.7847	0.6135	**0.0000**	**0.0000**	0.5101	0.2079	**0.0019**	**0.0000**
VM	0.4528	0.0867	**0.0000**	**0.0000**	0.3551	0.2391	**0.0039**	**0.0000**
BF	0.6847	0.1712	**0.0014**	**0.0000**	0.2615	0.1744	**0.0016**	**0.0000**
ST	0.3291	0.1341	**0.0000**	**0.0000**	0.3622	0.3445	**0.0120**	**0.0000**
LG	0.4885	0.0993	**0.0000**	**0.0000**	0.3879	**0.0150**	**0.0000**	**0.0000**
MG	0.5874	0.0806	**0.0000**	**0.0000**	0.3993	**0.0015**	**0.0000**	**0.0000**

The bold values indicate the statistical significance values of *p* < 0.05 with respect to RF.

## 4. Discussion

Human joint movement estimation using multi-channel sEMG signals has been widely used to enable HRI systems' intuitive and voluntary control. However, some issues have inhibited the collection of high-quality sEMG signals from all relevant muscles, such as weakness or spasticity of one or more specific muscles, mechanical/signal interference between EMG sensors and wearable robots/environment, discomfort for long-term use, etc. Therefore, using fewer channels or single-channel sEMG signals is of practical importance. It remains unknown whether the continuous knee joint movement can be estimated using single-channel sEMG signals. In addition, it is a challenge to ensure high estimation accuracy using only single-channel sEMG signals. This study verified the feasibility of continuous joint movement estimation only using single-channel sEMG signals and proposed a new feature extraction scheme, namely FG-CNN, to improve the estimation performance effectively.

The main advantage of FG-CNN is that it contains both handcrafted features and CNN features, which can improve the motion estimation performance just by using single-channel sEMG signals (as shown in Figure 5). When multi-channel sEMG signals were used, the handcrafted features or CNN features contained enough muscular information and could be successfully adopted in motion estimation. However, with the number of channels decreasing, the muscular information in the handcrafted features or CNN features will not be sufficient. To further extract implicit features, this study fed the handcrafted features into a fusion layer to guide the extraction of CNN features. Compared with both the handcrafted features and CNN features, the FG-CNN features can ensure a more accurate joint movement estimation, which implies that the FG-CNN features contained more muscular information.

**Figure 7:**
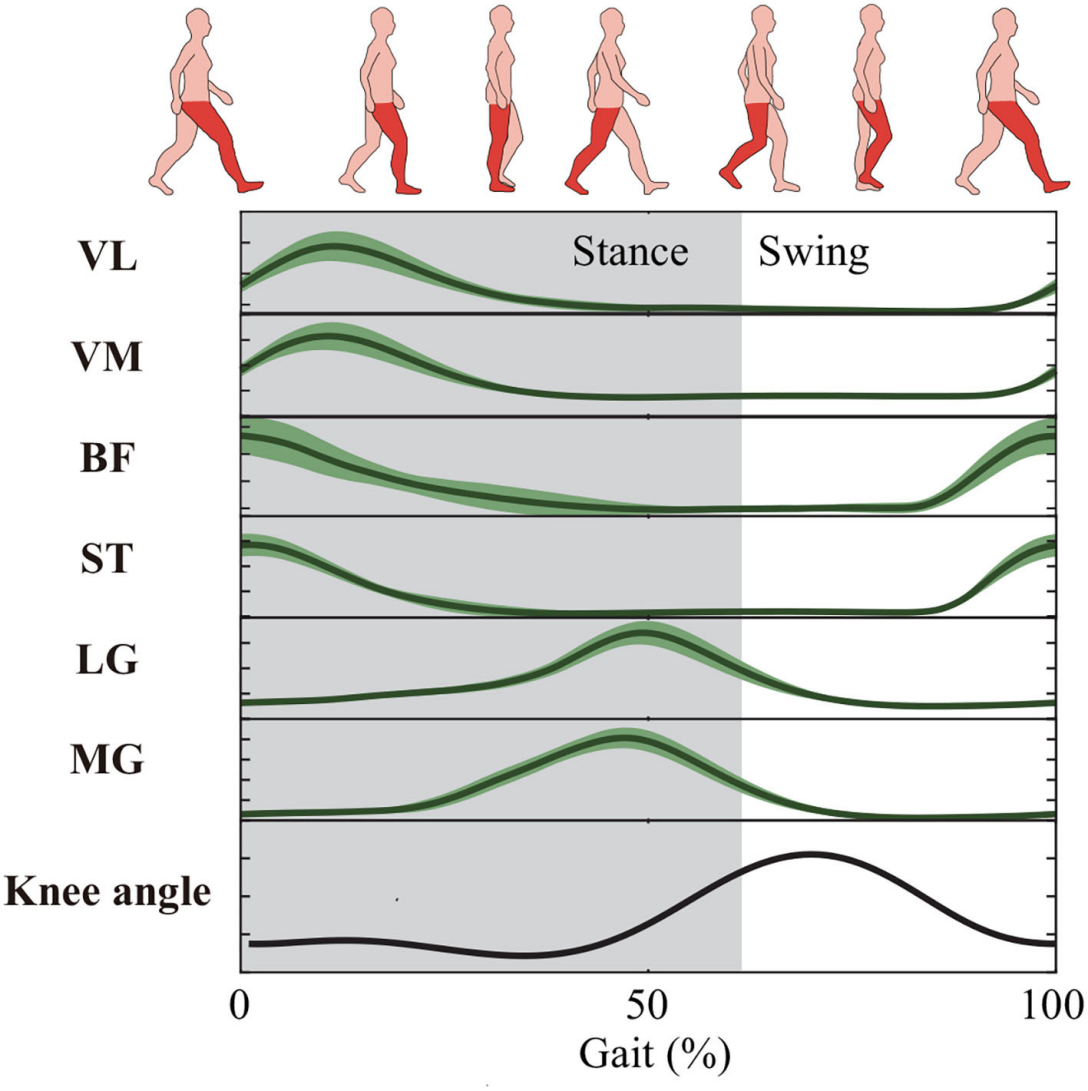
Muscle activity profiles and knee angle profiles during the gait cycle.

Based on the single-channel sEMG signals of LG or MG, the estimated angle profiles were similar to the reference during the gait cycle, as shown in Figure 3. On the contrary, for single-channel sEMG of VL, VM, BF, or ST, the trends of estimated angles profiles were reversed, especially at the stance phase. In this study, the used estimation methods, such as random forest regression and light gradient boosting machine, directly mapped the sEMG features to knee angles without any biomechanics. The estimation performance was influenced by the correlation between inputs (sEMG features) and outputs (knee angles). In a gait cycle, compared to the other four muscle activity profiles, the correlation between the gastrocnemius activity profiles and the knee angle profiles was stronger (shown in Figure 7). This can explain why the estimation accuracy from LG or MG is highest.

sEMG will be changed on different days and subjects due to the changing skin impedance, which is affected by physiological factors such as subcutaneous tissue, the physiological cross-sectional area of the muscle, or dynamic factors such as sweat. It is a complex and significant issue need to be addressed in practical application. To date, some studies made efforts to address this issue. Bao et al. established a two-stream CNN with shared weights to enhance inter-subject performances in the wrist kinematics estimation (Bao et al., 2021). The results showed that the NRMSE and CC values were 22% and 0.67, respectively, which outperformed a state-of-the-art transfer learning method. Dantas et al. demonstrated that the CNN decoder performed significantly better than polynomial Kalman filters in most analyzed cases of temporal separations (0–150 days) between the acquisition of the training and testing datasets (Dantas et al., 2019). The above studies demonstrated the potential for the utilization of CNN to address the limitations of using sEMG on different days and subjects. Therefore, although the proposed FG-CNN degrades its estimation performance using sEMG from different subjects or days, it is possible not to degrade too much. Future studies should advance in this direction.

In comparison with the previous studies, the main advantage of the proposed method is to achieve good estimation performance using single-channel sEMG signals rather than the multi-channels, which can be used to improve the usability of low limb wearable robotics in weakness or spasticity of one or more specific muscles (Wei et al., 2022). Although the mean CC values of the proposed FG-CNN with single-channel EMG signals were slightly lower than the CC values of the state-of-the-art studies with multi-channel sEMG (shown in Table 2), the mean CC values of the proposed FG-CNN is around 0.85, which suggests the strong correlation strength between the estimated and the measured.

**Table 2:** Comparison with related research.

**References**	**Number of sensors**	**Method**	**Performance (knee angle)**
Yi et al. (2022)	9	LSTM	CC = 0.88 ± 0.04
Zhong et al. (2022)	8	Muscle synergy-driven	CC = 0.92 ± 0.02
		ANFIS model	
Wang et al. (2021)	8	Multi-branch neural network	CC = 0.96 ± 0.03
**This work**	**1**	FG-CNN	CC = 0.858 ± 0.085 (LG)
			CC = 0.856 ± 0.057 (MG)

The bold values indicate the results of this work.

It is worth noting that the proposed methods have limitations. One method is that the sEMG data from each subject were recorded on the same day and the proposed FG-CNN was trained and tested on the same subject. This study did not consider the influence of sEMG changes on different days and subjects. Future studies should advance in this direction. Another method is that the estimation performance still has room for improvement. Future studies would be required to recruit more subjects and further to improve the accuracy of knee joint estimation by advanced single-channel sEMG-based methods.

## 5. Conclusion

In this study, a new feature extraction method, namely FG-CNN, was proposed to estimate human knee joint movement using single-channel sEMG signals. To verify the effectiveness of this method, sEMG signals measured from six muscles, including the vastus lateralis, the vastus medialis, the biceps femoris, the semitendinosus, the lateral or medial gastrocnemius (LG or MG), were separately evaluated for estimating knee joint movements using the proposed FG-CNN. The experimental results showed that combined handcrafted-CNN features outperform either the handcrafted features or the CNN features. In addition, the results demonstrated that the proposed FG-CNN method with sEMG signals from LG or MG can effectively estimate the movements with average NRMSE values of 15.2 ± 3.5% (LG) and 15.7 ± 3.1% (MG) and average CC values of 0.858 ± 0.085 (LG) and 0.856 ± 0.057 (MG). The performance of the proposed signal-channel sEMG-based FG-CNN method was comparable to those of traditional multi-channel sEMG-based methods. The proposed FG-CNN have the potential to provide an alternative means for knee joint movement estimation to overcome the aforementioned limitations faced by the traditional multi-channel sEMG-based methods.

## Data availability statement

The raw data supporting the conclusions of this article will be made available by the authors, without undue reservation.

## Ethics statement

The studies involving human participants were reviewed and approved by the local Ethics Committee of Nankai University. The patients/participants provided their written informed consent to participate in this study.

## Author contributions

WH, NY, and JH initiated and supervised the research project. SZ and JL carried out the research. SZ analyzed the data. SZ, JL, WH, NY, and JH interpreted the data and drafted and revised the manuscript. All authors read and approved the final manuscript.

## Funding

This work was supported by the National Natural Science Foundation of China (U1913208, 61873135, and 61720106012).

## Conflict of interest

The authors declare that the research was conducted in the absence of any commercial or financial relationships that could be construed as a potential conflict of interest.

## Publisher's note

All claims expressed in this article are solely those of the authors and do not necessarily represent those of their affiliated organizations, or those of the publisher, the editors and the reviewers. Any product that may be evaluated in this article, or claim that may be made by its manufacturer, is not guaranteed or endorsed by the publisher.
